# Spatial and temporal patterns of porcine reproductive and respiratory syndrome virus (PRRSV) genotypes in Ontario, Canada, 2004–2007

**DOI:** 10.1186/1746-6148-10-83

**Published:** 2014-04-05

**Authors:** Thomas Rosendal, Cate Dewey, Robert Friendship, Sarah Wootton, Beth Young, Zvonimir Poljak

**Affiliations:** 1 Department of Population Medicine, University of Guelph, Guelph, ON, Canada; 2 Department of Pathobiology, University of Guelph, Guelph, ON, Canada

**Keywords:** PRRSV, Area spread, Cluster, Clustering, Aerosol, Swine, Pig

## Abstract

**Background:**

The spread of PRRSV among pig herds has been investigated experimentally, but few observational studies have investigated this subject. Because PRRSV is endemic and live modified vaccines are used in Ontario, the spatial and temporal distributions of 6 PRRSV genotypes were investigated in the province during the period from 2004–2007. The purpose was to find evidence of spread of PRRSV genotypes and determine if spread could be attributed to supplier or ownership connections between herds. Sequence information from PRRSV ORF5 and related source-herd demographic information were obtained from diagnostic submissions to the Animal Health Laboratory, University of Guelph.

**Results:**

A spatial cluster that could not be attributed to supplier or ownership connections among herds in the cluster was detected for RFLP type 1-3-4. Because of genetic dissimilarity among members of the cluster, it was considered to be a result of past spread of the RFLP type. A spatio-temporal cluster detected for RFLP type 1-18-4 was attributed to a shared gilt supplier among the herds in the cluster. Significant spatio-temporal patterns detected for RFLP type 2-5-2, which is considered to be a vaccine-type virus were most likely due to grouping of herds in an ownership that used the corresponding vaccine. Clustering within herd-ownership was a risk factor for presence of five of the six genotypes investigated in the present study.

**Conclusions:**

Although the literature indicates that PRRSV can spread via aerosol between pig herds, the present study found no strong evidence of this occurring in Ontario. The evidence pointed toward transmission of PRRSV occurring in this population by common sources of animals or similarity of herd ownership, which is a proxy measure for other connections between herds. It is also apparent that the recognition and testing of these connections between herds is a necessary part of interpreting spatio-temporal patterns of PRRSV genotypes.

## Background

Since its emergence in North America in 1987 [1], porcine reproductive and respiratory syndrome virus (PRRSV) has been one of the most important swine pathogens. Worldwide costs due to PRRSV have been ranked as the third largest contributor to losses in swine populations in the period between 2006 and 2009 [2]. In the United States alone, annual PRRSV losses were estimated at $560 million in 2004 [3], and in Ontario, Canada, costs have been estimated at $36-$73 million per year [4]. Considering the importance of this virus, surprisingly little observational data about PRRSV spread is currently available. Information is even scarcer about spatial distribution of specific genotypes of PRRSV. The spatial and temporal distribution of PRRSV in Ontario, might best be investigated at the genotype level rather than the virus level for at least two reasons. First, PRRSV is an endemic pathogen in this swine population [5,6], but new PRRSV genotypes continue to emerge and spread among herds. Second, common use of modified live PRRSV vaccines in the source pig population makes the definition of infection status by genotype more attractive than simply identifying the presence or absence of PRRSV in a herd. Multiple genotypes of PRRSV, as described by restriction fragment length polymorphism (RFLP), occur in Ontario [7,8]. The spatial and temporal distribution of these genotypes in Ontario has not previously been described. Variation of PRRSV genotypes in space and time may indicate area spread of the virus, ie, transmission of the virus among herds proximal in space due to aerosol transmission or other factors clustered in space. A simulation of area spread by aerosol transmission of PRRSV has been demonstrated experimentally by forcing air from a space with PRRSV-infected pigs into one with PRRSV-negative pigs [9,10]. It has also been demonstrated that PRRSV can infect pigs in a structure a short distance (120 m) downwind from a structure housing PRRSV-positive pigs [11]. More distant downwind aerosol dissemination, up to 9.1 km from an infected herd, has also been demonstrated [12]. The ability of PRRSV to spread by aerosol may vary by genotype [13], and therefore we might expect to see that spatiotemporal patterns under field conditions differ by genotype. Where area spread occurs, PRRSV isolates of the same genotype might be closer in space and time than different genotypes. The relationship between spatiotemporal proximity and homology of PRRSV open reading frame 5 (ORF5) was investigated in Illinois, where no significant correlation was found [14], and in Minnesota, where a significant spatial distance-genetic homology correlation was reported [15]. Direct aerosol transmission is only one of several events that would create spatiotemporal patterns in PRRSV genotype distribution. Herds under the same ownership might be clustered in space, and sharing management practices, personnel, and animals would result in a similar PRRSV genotype among those herds. Herds proximal to each other but under different ownership may share common suppliers of animal, semen [16,17], and feed or equipment, all of which could facilitate transmission of PRRSV [18].

The objective of this study was to investigate patterns in PRRSV distribution that could indicate area spread of PRRSV. Specifically, to assess the spatial and the temporal patterns of 6 common PRRSV RFLP types detected in Ontario swine herds between 2004 and 2007, and to assess the degree to which ownership structure influenced the observed patterns. For this publication, area spread is defined as transmission of PRRSV from herd to herd in space by unknown factors, which may include aerosol transmission. Aerosol spread is defined as transmission of PRRSV from herd to herd by a virus travelling through the air. In this study, aerosol spread cannot be distinguished from more general area spread.

## Methods

### Herd inclusion

Study design and sample collection are described in detail in a previous report [7]. Briefly, samples from PRRSV-positive submissions to the Animal Health Laboratory at the University of Guelph were eligible for inclusion from September 1, 2004 to August 31, 2007. For each submission, the submitting veterinarian and herd owner were contacted to determine if the owner was willing to participate. The date of sample collection from the herd was identified from the diagnostic-sample submission report. A telephone survey was administered to each herd owner or manager with questions about herd demographics, herd location, and ownership structure. An owner of a herd could be an individual or a corporation and could own multiple premises or a single premises. A herd was included only if a sample from the submitting premises had not been previously included in the study. Complete information was available from 307 premises.

### Genetic analysis and virus classification

The open reading frame 5 (ORF5) gene of PRRSV was sequenced for all samples included in the study. The RFLP type, based on the University of Minnesota expanded nomenclature [19], which is an extension of the Wesley nomenclature [20], was determined from the sequence data. The 3 most common wild-type virus RFLP types (1-3-4, 1-18-4, and 1-8-2) were selected for further analysis. Restriction fragment length polymorphism type 1-8-4, occurring in only 7 cases, was also included for analysis, because this virus type emerged in Ontario during the study period [7] This RFLP type is also of interest because an isolate of RFLP type 1-8-4 known as MN184 has been reported to be more likely to be transmitted by aerosol than a less pathogenic type [13]. This genotype was also the only one of 3 PRRSV RFLP types tested in a previous experimental study to be transmitted by aerosol out to 9.1 km from an infected herd [12]. The 2 RFLP types considered consistent with vaccine strains 2-5-2 and 1-4-2 were also included in further analyses.

### Data analysis

Data were analyzed to determine if spatial and temporal trends or clustering were present for each of the 6 genotypes of interest. For each analysis, a herd positive for one of these 6 genotypes was defined as the case, and herds positive for other RFLP types were defined as non-cases. In this way, the heterogeneity of the total herd distribution should not result in the detection of clustering where none was present. The spatial distribution of cases and non-cases for a genotype was first visualized as a point map and as a smoothed risk-map, using parametric kernel smoothing in order to uncover the presence of obvious trends. Kernel smoothing was performed using the quartic kernel with equal bandwidth parameters of 25 km in both the x and y directions in *R* version 2.10.0 (http://www.r-project.org) using the *splancs* library (http://cran.r-project.org/web/packages/splancs/index.html). Spatial autocorrelation was investigated using the D-function, which illustrates excess spatial clustering of cases versus non-cases [21]. The simulated *p*-value for determining spatial autocorrelation in the D-function was assessed over 100 km of spatial separation. Autocorrelation of each RFLP type in both space and time was visualized using the space-time K-function [22]. Autocorrelation in space-time was tested over a range of 20 km and 90 days using a simulated *p*-value. A proportional plot of the K-statistic over space and time was also visualized, and a K-statistic > 1, was considered to indicate positive aggregation of cases at a distance in space-time. The existence and location of spatial clusters were assessed by the spatial scan statistic using the Bernoulli model and default parameters available through the SaTScan v9.0.1 64-bit software package (http://www.satscan.org). Similarly, the existence, location, and period of space-time clusters were evaluated using the space-time permutation model in the same software. Cluster detection analysis was performed using 999 simulations.

As a spatial cluster might exist if its members shared common ownership or suppliers, the members of each cluster were assessed using descriptive statistics and simple cross-tabulations of their recorded covariates. In addition, herds included in the significant clusters identified by the spatial scan statistic or the space-time permutation model were further investigated by more detailed genetic analysis of PRRSV ORF5 detected in those herds. All members of the genotype of interest where included in a dendrogram generated by the unweighted pair group method with arithmetic mean (UPGMA). Close grouping of members of a spatial or space-time cluster on the dendrogram was interpreted as evidence of connections among the herds in the cluster. For example, one might find a cluster of cases of an RFLP type and then ask the question “Why is there a cluster of cases?” Step 1 was to determine whether the herds shared common animal suppliers, ownership, semen suppliers, etc. If no commonalities were found then, step 2 was to determine whether the viruses in the spatial or space-time cluster were more genetically similar to one another than to the other members of the RFLP type. If that was true, it indicated idiopathic area spread. In addition to significant clusters, investigation of all primary spatial and space-time clusters and 2 secondary clusters for the common field RFLP types was performed if the radius of a cluster was < 20 km for clusters with ≥ 3 herds and < 10 km for clusters with only 2 herds. This investigation was performed by searching for common links among cluster herds, regardless of the statistical significance of the clusters, with the rationale that area spread could occur among a small number of herds, which could be difficult to identify as a significant cluster.

Trends in space and time were also modeled using the generalized additive model (GAM) in *R* using the MGCV (http://cran.r-project.org/web/packages/mgcv/index.html) library version 1.6-1. Generalized additive models included parametric terms, and non-parametric smoothing terms for space and time using thin plate regression splines [23]. The fitting process and the method used to prevent overfitting are described elsewhere [23]. The model output for the non-parametric terms includes an indication of the model “wiggle” expressed as estimated degrees of freedom (EDF). A larger value of EDF suggests a more complex surface. In addition, the model output for the non-parametric terms containing spatial effect was evaluated as a map on a probability scale, interpreted as an expected proportion of a particular RFLP in the population on a fine grid. A decision was made to test the effect of ownership structure as a fixed effect in a parametric part of the GAM model: each large owner (owners with ≥ 5 different premises that had submitted samples to the database) was compared to all small ownerships (those that had submitted samples from < 5 different premises) as the referent group. Six ownerships had ≥ 5 different premises that submitted samples: these owners are identified as Owners 1 through 6 (Table 1). A model building approach was used to evaluate the statistical significance of both parametric and non-parametric variables in univariable models. This was followed by an attempt to build multivariable models to evaluate the association of each RFLP type with time, geographical space, and ownership. In addition, for each PRRSV RFLP type, space-time interaction in GAM was assessed by evaluating the statistical significance of a tensor product smooth of the space and time variables, as previously described by Simon Wood in 2006 [24].

**Table 1:** Frequency of PRRSV RFLP types, tabulated by owner-identifier in Ontario 2004–2007

	**Wild-type viruses**				**Vaccine-type viruses**		**Total**
**RFLP owner**	**1-3-4**	**1-18-4**	**1-8-2**	**1-8-4**	**2-5-2**	**1-4-2**	
Small owners	31	25	16	4	36	14	247
1	1	5	0	0	7	0	14
2	2	1	3	2	1	1	12
3	5	5	0	0	0	0	11
4	0	0	0	1	0	0	10
5	0	3	0	0	2	0	7
6	0	0	0	0	4	0	6
**Total premises**	**39**	**39**	**19**	**7**	**50**	**15**	**307**

## Results

**Figure 1:**
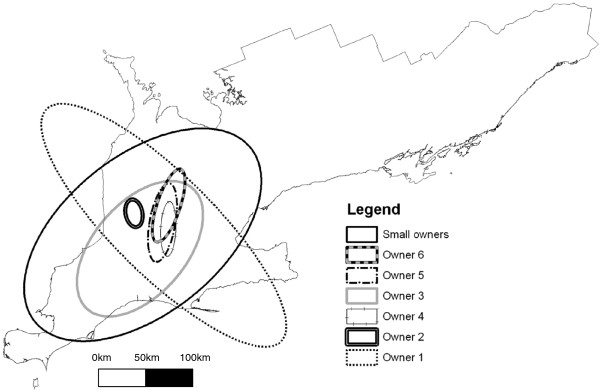
**Ellipses representing the locations of the large owners in the data.** Each ellipse represents the location of 95% of the point locations of herds within each ownership. Owner 1: 14 sites; Owner 2: 12 sites; Owner 3: 11 sites; Owner 4: 10 sites; Owner 5: 7 sites; Owner 6: 6 sites; Small owners (fewer than 4 sites per ownership): 247 sites.

Three hundred and seven (307) submissions to the Animal Health Laboratory from 221 owners were included in the study. The number of included premises within each of the 6 largest ownerships in the dataset are listed in Table 1. Of the small ownerships, 6 owners were each represented 3 times, 20 owners were each represented twice, and the remaining 189 owners were each represented only once in the data. The geographic distribution of the ownerships included in the analysis are illustrated by 95% ellipsoids in Figure 1 in order to show the distribution of ownerships without displaying actual herd locations. The frequency of the genotypes included in the analysis are listed in Table 1. In total, 39 RFLP types were identified. Including those in Table 1, eight RFLP types were represented > 9 times, eight were represented 6 to 9 times, seven were represented 2 to 5 times, and sixteen were represented only once in the data. The frequency of each genotype by herd ownership is displayed in Table 1. Results of statistical testing for each analysis are summarized in Table 2.

**Table 2:** **Summary of *p*-values for tests of spatial and space-time clustering and clusters, and epidemiological measures of association between occurrence of PRRSV RFLP types and time, space, and ownership in Ontario from 2004–2007**

	**Wild-type viruses**				**Vaccine-type viruses**	
**RFLP type**	**1-3-4**	**1-18-4**	**1-8-2**	**1-8-4**	**2-5-2**	**1-4-2**
	*Tests and functions for spatial and space-time clusters and clustering (p-values)*					
D-function	0.06	0.18	0.35	0.37	0.94	0.11
Space-time *K-* function	0.63	0.06	0.10	0.86	**0.005**	0.48
Spatial scan statistic^**a**^ (Bernoulli model)	**0.012**	0.41	0.22	0.32	0.09	0.60
Spatial scan statistic^**a**^ (Space-time perm. mod.)	0.13	**0.08**	0.72	0.99	**0.04**	0.17
	*Univariable generalized additive models (p-value and OR*^**b**^*for individual ownerships)*					
Space (x,y) (*p*-value)	0.81	**0.01**	0.71	0.40	0.51	0.94
Time (days) (*p*-value)	**0.008**	0.20	0.80	0.62	0.61	0.06
Ownership	**3: OR = 4.1**	**1: OR = 4.9**	**2: OR = 4.8**	**2: OR = 12.1**	**1: OR = 5.9**	NS^**c**^
Baseline = small owners	**4: OR = 7.3**	**3: OR = 7.4**			**6: OR = 11.7**	
	**5: OR = 12.2**	**5: OR = 6.7**				
	*Final multivariable generalized additive models (p-value and OR*^**b**^*for individual ownerships)*					
Space (x,y) (*p*-value)	NI^**d**^	**0.007**	NI	NI	NI	NI
Time (days) (*p*-value)	**0.008**	NI	NI	NI	NI	**0.059**
Ownership	**3: OR = 6.2**	**1: OR = 5.7**	**2: OR = 4.8**	**2: OR = 12.1**	**1: OR = 5.9**	NI
Baseline = small owners		**3: OR = 10.3**			**6: OR = 11.7**	
		**5: OR = 15.5**				

^**a**^*p*-values obtained for the primary spatial and space-time cluster detected by this method.

^**b**^OR = Odds Ratio for individual ownerships using small owners as the baseline. Only comparisons with *p* < 0.05 are listed in the table. Number before OR corresponds to the ownership code as listed in Table 1.

^**c**^NS = No comparisons were significant at *p* < 0.05.

^**d**^NI = Not included in the final model.

Boldface values have *p*-values < 0.05.

### RFLP type 1-3-4

**Figure 2:**
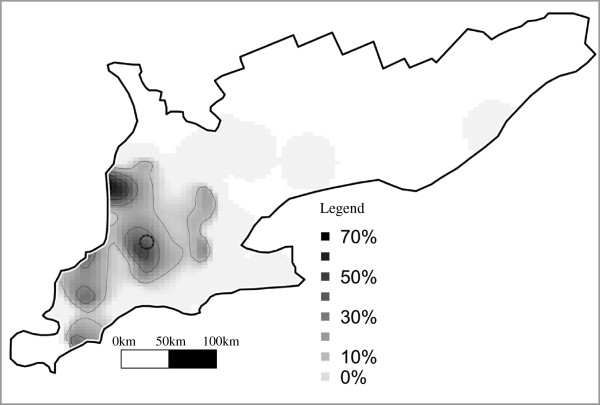
Risk map generated by kernel smoothing of RFLP type 1-3-4, and the cluster identified by the spatial scan statistic indicated by a dashed ring on the map.

**Figure 3:**
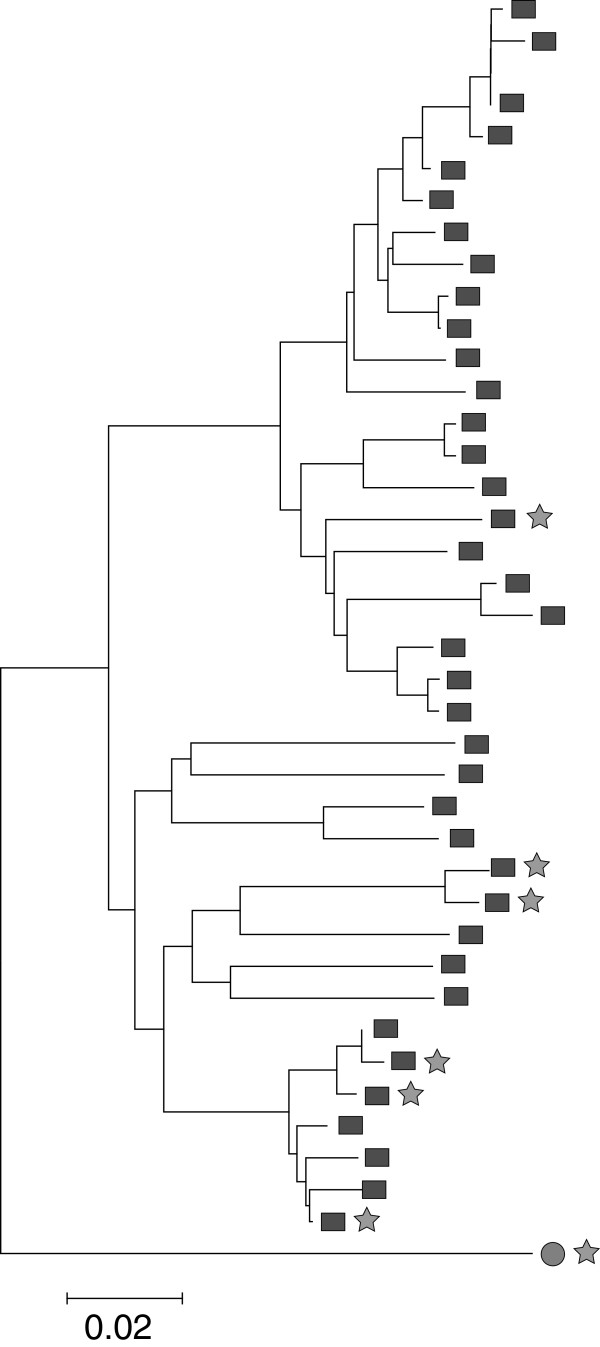
**An unweighted pair group method with arithmetic mean (UPGMA) dendrogram of all members of RFLP type 1-3-4 and those members of the spatial cluster detected in RFLP type 1-3-4.** Solid grey rectangles indicate those viruses that are members RFLP type 1-3-4. Members of the spatial cluster are labeled with grey stars. A single vaccine-type virus was a member of the spatial cluster and is labeled at the bottom of the dendrogram with a solid grey circle and a grey star. The scale is in fraction of nucleotide dissimilarity over the ORF5 gene of PRRSV.

By kernel smoothing, RFLP type 1-3-4 showed an uneven distribution (Figure 2). A significant spatial cluster with a radius of 8.1 km was detected (RR = 7.8, *p* = 0.015), including 7 herds, of which 6 were cases. The location of this cluster is indicated by a dashed ring in the map in Figure 2. The herds in the cluster were: a 45-sow farrow-to-finish operation, a 1100-head finisher barn, an all-in all-out by site 2000-head finisher barn, a 1275-sow farrow-to-grower operation, a 650-sow farrow-to-grower operation, and a 2250-sow farrowing barn. The membership of the cluster did not share ownership, boar sources, or gilt sources, but 2 members used the same semen source. A dendrogram of all members of RFLP type 1-3-4 and the viruses within the cluster is displayed in Figure 3.

**Figure 4:**
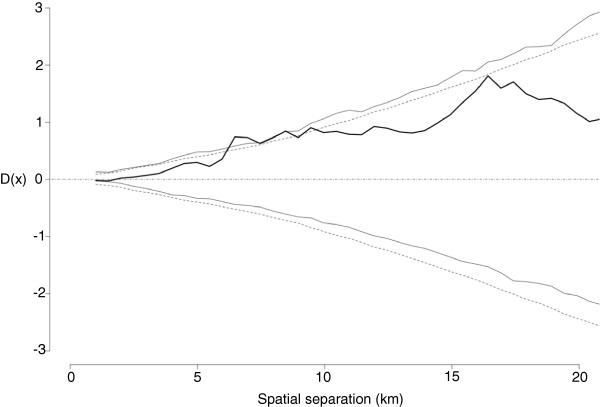
**Illustration of the lack of spatial autocorrelation of RFLP-type 1-3-4 by the D-function over a 20 km distance.** The solid black line is the point estimate of the D-function, the dotted grey line is the approximated 95% confidence interval, the solid grey line is the simulated 95% confidence interval.

The D-function for RFLP type 1-3-4 did not strongly suggest spatial aggregation of case herds (Figure 4). It is provided here as it was the strongest indication of spatial clustering for each of the genotypes evaluated, both by visual assessment and by the formal test (Table 2, *p* = 0.06).

**Figure 5:**
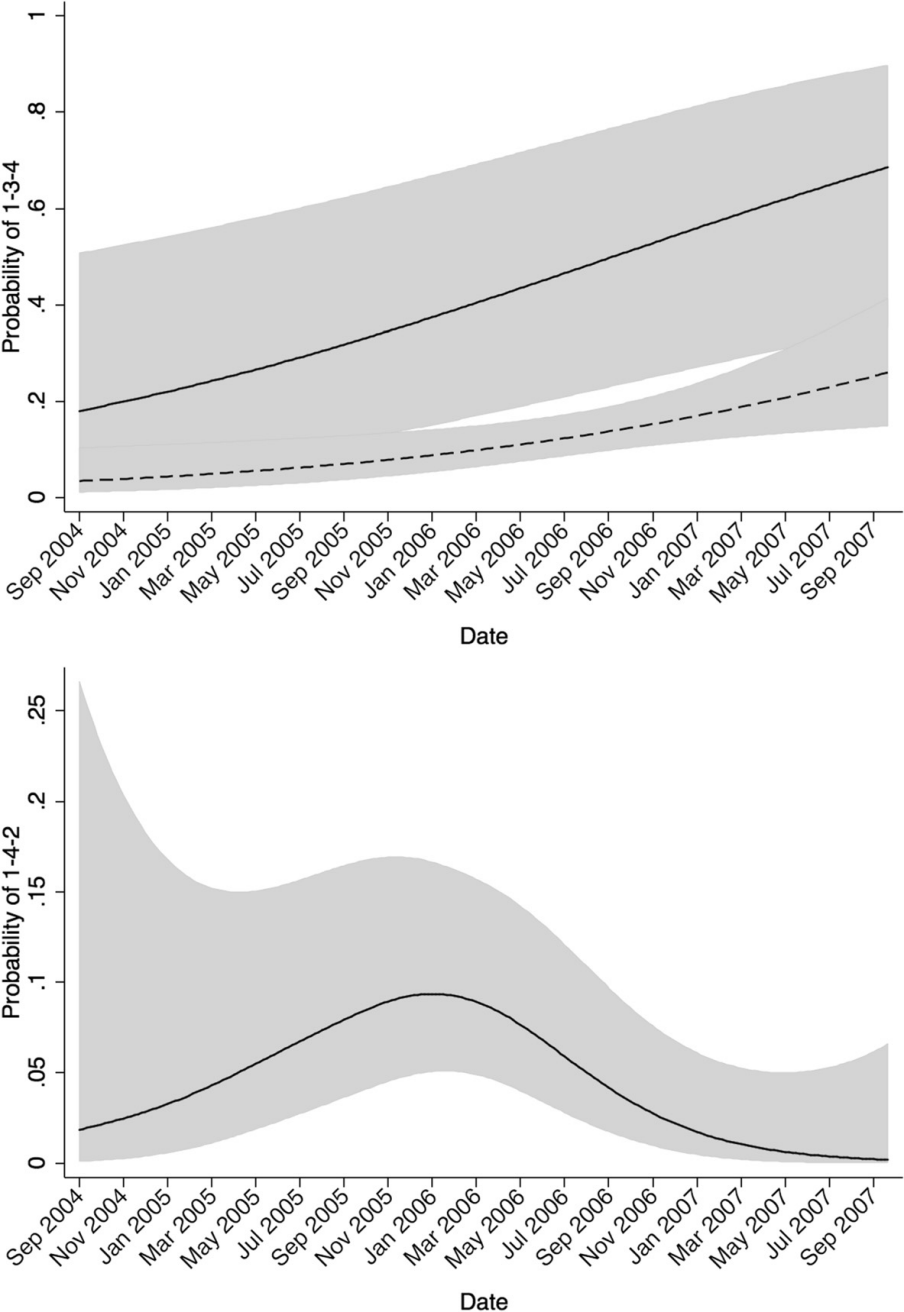
**Predicted probability of herds being infected with PRRSV for 2 RFLP types in Ontario from 2004–2007.** The top graph illustrates the model predicted probability of RFLP type 1-3-4 for small owners (dashed line) and for large owner 3 (solid line) and the associated 95% confidence intervals surrounding each (grey zone). The bottom graph illustrates the model predicted probability of RFLP type 1-4-2 (black line) and the 95% confidence interval surrounding it (grey zone).

A temporal trend found in RFLP type 1-3-4 using the GAM has been previously described [7]. This trend was also present after accounting for ownership in the GAM (EDF = 1) (Table 2, Figure 5).

### RFLP type 1-18-4

**Figure 6:**
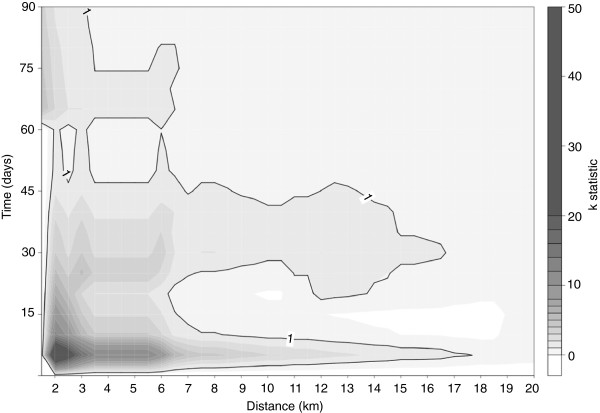
**Space-time K-function for the range of 90 days and 20 km for PRRSV RFLP type 1-18-4 in Ontario from 2004–2007.** The black boundary line on the graph indicates where the K-statistic = 1.

Kernel smoothing of Minnesota RFLP type 1-18-4 suggested a high probability region in the northwestern part of the study area (data not shown). The space-time K-function for type 1-18-4 indicated a trend toward spatiotemporal autocorrelation (Table 2, Figure 6). The residuals of the space-time K-function tended to be > 0, with a range of -1 to 6, showing a decreasing pattern over the range of the K-statistic which could be interpreted as evidence of spatial-temporal clustering for this genotype. The relative plot of the space-time K-function (Figure 6) was consistently > 1 over a spatial distance of up to ~ 6 km and temporal distance of ≤ 45 days. In concordance with this, a space-time cluster was identified for this genotype (*p* = 0.08), with a radius of ~11.5 km during 27 days in October 2006. It contained 3 herds: 2 farrow-to-finish herds and 1 farrow-to-grow herd. All 3 herds were supplied by the same gilt source and had the same veterinarian, and 2 had the same semen source. Once these 3 herds were omitted from the analysis, the space-time K-function evaluated on remaining cases for this RFLP type gave no indication of space-time clustering.

**Figure 7:**
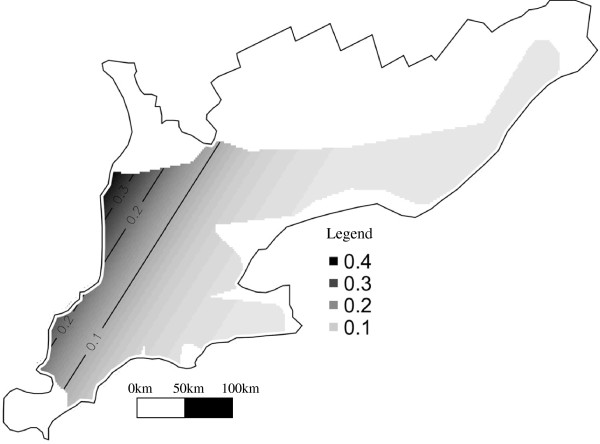
Risk map for PRRSV RFLP type 1-18-4 in Ontario from 2004–2007 generated from the GAM predicted probability of type 1-18-4 for small ownership herds.

Generalized additive models indicated a significant spatial trend for type 1-18-4 (EDF = 2) (Table 2). A higher expected probability for this genotype in the northwest part of the study area was found and this spatial trend remained significant after accounting for ownership (Table 2 and Figure 7). Owners 1, 3, and 5 had significantly increased risk of having type 1-18-4 than did small owners (Table 2). No interaction between the spatial and the temporal term could be detected for this genotype, indicating that the simple spatial trend did not significantly change over time during the study period.

### RFLP type 1-8-2

Kernel smoothing indicated a higher proportion of Minnesota RFLP type 1-8-2 in the northwestern part of the study area. Ownership was significantly associated with the presence of this genotype. The single large owner with genotype 1-8-2 (Table 1) was at increased risk of being positive for this RFLP type (Table 1, 2).

### RFLP type 1-8-4

**Figure 8:**
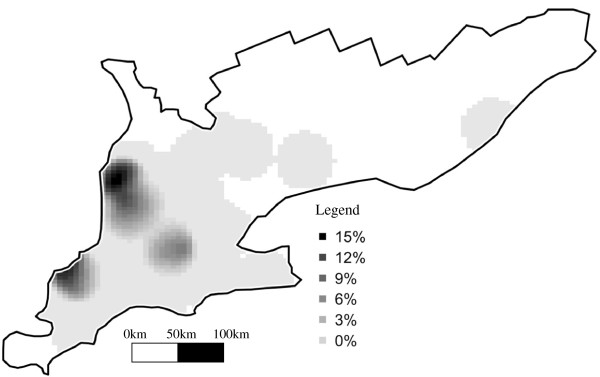
Kernel smoothing risk map of PRRSV RFLP type 1-8-4 in Ontario from 2004–2007.

Kernel smoothing of Minnesota RFLP type 1-8-4 suggested 3 distinct areas at increased risk for this strain (Figure 8). However, no significant spatial or spatio-temporal clusters could be detected by the methods utilized in this study. One large ownership was at significantly greater odds of having this genotype (Table 2).

### RFLP type 1-4-2 (vaccine-like strain)

There was a significant temporal effect in RFLP type 1-4-2 (Table 2, Figure 5) (EDF = 2.2). This genotype is considered to be consistent with the Boeringer-Ingelheim Ingelvac PRRS ATP™ vaccine type [8,20,25], which was licensed for use in Canada in 2000 [26]. This vaccine was used in 24 small ownership herds and 2 herds belonging to Owner-2. No significant clustering and no clusters could be detected for this strain.

### RFLP type 2-5-2 (vaccine-like strain)

The RFLP type considered to be consistent with the Boeringer-Ingelheim Ingelvac PRRS MLV vaccine is type 2-5-2 [8,20,25]. This vaccine was licensed for use in Canada in October 1997 [26]. Kernel smoothing revealed a higher-risk area for RFLP type 2-5-2 in the southeastern part of the study area. The space-time K-function indicated a degree of temporal autocorrelation in a geographic range between 5 and 20 km (Table 2). The residuals from the space-time K-function ranged from 0.5 to 7 and remained centered on 2 across values of the K-statistic. This was consistent with evidence of the space-time aggregation. However, a minimum spatial distance of 5 km was not in concordance with area spread, suggesting some other mechanism of spatial aggregation. Similarly, a significant spatio-temporal cluster of this strain was detected (*p* = 0.04; Table 2). This cluster contained 4 herds, with a radius of approximately 25 km, duration 153 days, starting in January 2005. This cluster was in part driven by ownership: 2 finisher herds belonged to the same system, and the other 2 herds also belonged to 2 large production systems.

Two large ownerships, 1 and 6, were at significantly greater odds of having this genotype than were small ownerships (Table 2). However, no significant spatial trend could be detected by GAM. The Ingelvac PRRS MLV vaccine was used in 93 small ownership herds, two herds in Ownership-1, two herds in Ownership-3, and three herds in Ownership-4.

### Investigation of non-significant clusters

Of the 9 most likely purely spatial clusters for wild-type PRRSV genotypes (two for type 1-3-4, three for type 1-18-4, two for type 1-8-2, and two for type 1-8-4), four were considered too geographically widespread (> 20 km radius) to be a likely result of local spread. Of the remaining 5, one cluster was possibly due to spread through the common gilt source (1-18-4) and one cluster was most likely due to spatial aggregation of herds under one ownership (1-8-2). In 2 other clusters there was no obvious link except geographical space (1-8-2 and 1-3-4), and for 1 cluster there was a common link for destination of culled sows and geographical space (1-18-4). Beyond these 9 most likely clusters, some small clusters (n = 2 herds) were detected that consisted of herds of different ownerships with RFLP types that were identical or similar to those in herds outside these clusters. In 1 small cluster of RFLP type 1-3-4, it was plausible from a temporal and biological perspective to assume that local spread could have occurred from a nursery to a sow herd. In another small cluster of RFLP type 1-3-2, such local spread could not be inferred from a biological and temporal perspective.

Of 10 non-significant space-time clusters for wild-type PRRSV genotypes (three for type 1-3-4, two for type 1-18-4, three for type 1-8-2, and two for type 1-8-4), five were considered to be too geographically widespread (> 20 km radius) to be a likely result of local spread. Among the remaining five clusters, two included herds with no apparent links except geographical proximity (types 1-3-4 and 1-18-4), two clusters were each formed exclusively of herds belonging to the same large ownership (type 1-8-2), and one small cluster (n = 2) that were within a radius of 11 km and shared a common semen source. The number of herds in these five clusters varied from 2 to 5, the radius varied from 2 to 18 km, and the duration varied from 6 to 209 days.

## Discussion

Current knowledge about spread of PRRSV among herds has been gathered from different sources, including experimental models [10,12,27-30] and informal outbreak investigations [17,31,32]. Epidemiological investigations of PRRSV spread with a time component are relatively infrequent [33,34]. In a study of herds that were initially PRRSV-negative [33], Holtkamp et al. reported that biosecurity factors were associated with the length of time to the first outbreak of PRRS. In a second study, Mortensen et al. investigated the spread of a North American PRRSV type in Danish pig-herds, some of which had already been exposed to the distinctly different European PRRSV type [34]. In contrast, the purpose of our present investigation, was to summarize patterns of spread of different endemic PRRSV strains in a population of herds where there was no clear outbreak of a novel virus that was the subject of the investigation. The study was motivated in part by the earlier finding that the ability of PRRSV strains to spread through air is strain dependent, which might contribute to some genotypes exhibiting evidence of local spread while others do not [13].

### RFLP type 1-3-4

Type 1-3-4 was the only strain for which the existence of a significant spatial cluster was detected. As the origin of the type 1-3-4 spatial cluster could not be explained by commonalities in the recorded sources of animals entering the herds or ownership among the herds, further molecular analysis was performed. If the members of the spatial cluster also clustered genetically within the already related group of RFLP type 1-3-4, it was hypothesized that this spatial cluster existed because of virus spread among members of the cluster. In the case of a recent outbreak, one would expect that members of a spatial cluster are genetically closely related, and therefore closely grouped on the dendrogram. However, the dendrogram suggested such relatedness for only a limited number of PRRSV isolates, suggesting that the spatial cluster was most likely not due to recent PRRSV spread from a common source. Virus type 1-3-4 has previously been reported to be associated with higher than expected preweaning mortality [7], and it has been shown that a higher pathogenicity PRRSV isolate is more likely to spread by aerosol [13]. Thus, further investigation is warranted to determine the ability of RFLP type 1-3-4 to spread, relative to other PRRSV genotypes.

The final GAM model illustrated in Figure 5 shows that, after accounting for large ownership structure, there is still an increase in RFLP type 1-3-4 over the study period. The risk for Owner 3 was numerically higher than that for small owners throughout the study period, but was significantly higher only from January 2006 to April 2007 (Figure 5). A previous report utilizing the same data without adjusting for ownership showed a similar trend [7]. This increase over the entire study period may be an indication that this genotype was slowly emerging in Ontario, during and after the study period. Because its frequency was increasing during the study, the need to further investigate how RFLP type 1-3-4 can spread is reinforced.

### RFLP type 1-18-4

The spatial pattern of this genotype indicates a trend of high to low prevalence from northwest to southeast in an almost linear fashion, which remains apparent after adjusting for ownership in the data (Figure 7). The model coefficients in Table 2 also reveal the relative importance of the spatial effect, when compared to the ownership effect. The model indicates that 10%-30% of herds from small owners were expected to be positive for 1-18-4, in contrast to a 90% expected probability of a large ownership herd in the northwest being positive. The trend was greater in areas adjacent to lakes in the north and west, indicating that the 1-18-4 genotype either emerged in this region or was introduced in a comparatively higher proportion of herds in this area by long-distance transport from other areas. The spatial trend that remained after adjusting for ownership may be a result of direct spread from herd to herd across the study area by aerosol or by unknown connections between herds. Another possibility is that multiple study herds were infected from a common source such as a semen or animal supplier.

The space-time K-function of RFLP-type 1-18-4 indicated a tendency for space-time autocorrelation in the range of < 45 days and < 6.5 km. This was the only evidence of area spread that could be detected by the methods employed in this study, and the tendency was further confirmed by detection of a space-time cluster with similar dimensions in space and time as the range of the autocorrelation detected by the K-function. The range of this potential spatial spread is also in concordance with previous experimental findings indicating that PRRSV may spread as far as 9.1 km [12]. However, because of a shared gilt supplier among herds in the cluster, movement of gilts from the same supplier was most likely responsible for this space-time cluster. Also, the removal of the cluster from analysis resulted in a non-significant space-time K-function. For RFLP type 1-18-4, the superficial appearance of spread linked in space and time could be explained by the more plausible common animal source among herds.

### RFLP type 1-8-2

No significant spatial or temporal patterns were detected in this genotype. The most important factor was that a large owner (Owner 2) was at significantly increased risk.

### RFLP type 1-8-4

Because our data contained only 7 occurrences of this genotype, the results of the analysis should be interpreted cautiously. Conversely, the fact that no significant spatial or temporal trends or clusters were discovered, although both appeared in the raw data, could also be due to the small number of observations. The hot spots that were apparent in the kernel smoothing of RFLP type 1-8-4, but were not analytically corroborated, nevertheless may have been of biological importance. Owner 2 was at increased risk of this genotype and its member herds were highly clustered in space. One of the 3 hot spots in the kernel smooth risk map corresponds to the location of this owner. An examination of the point location of RFLP type 1-8-4 cases indicated that the hot spot included 2 cases from Owner-2 and 1 case from another owner proximal in space to Owner-2. This result could be because Owner-2’s herds were responsible for an outbreak which infected another local herd or because Owner 2 was affected by an outbreak occurring in the area. Temporal order of the cases cannot help us to understand directionality of spread, because it is unknown how many cases missing from the data were part of the hypothetical outbreak. A previous investigation of PRRSV in Ontario from 1998–2000 did not identify RFLP type 1-8-4 [8], but in a Quebec study with samples collected from 1998 to 2002, the genotype was identified more frequently than any other (28%) [25]. A previous phylogenetic investigation of an outbreak of RFLP type 1-8-4 in Minnesota in 2001 suggested that the origin of the Minnesota outbreak was Quebec [35]. Because Quebec neighbours Ontario, it also seems plausible that the genotype originated in Quebec and spread to Ontario. However, the spatial distribution does not support this argument, given the tendency toward a higher frequency of 1-8-4 in the western part of the study region, not adjacent to Quebec (Figure 8). If RFLP type 1-8-4 did spread from Quebec to Ontario, then it is possible that, given the ownership effect, the spread occurred through shipments of pigs from Quebec to specific owners in Ontario distant from the Quebec border. The present observational study could not contribute supporting field-evidence of the previous indications that RFLP type 1-8-4 is more likely, than some other PRRSV genotypes to spread by aerosol [12,13].

### Vaccine-like RFLP type 2-5-2; RFLP type 1-4-2

The GAM model did not confirm the presence of the apparent spatial aggregation in the kernel smoothing of RFLP type 2-5-2. However, the space-time K-function indicated space-time autocorrelation, possibly because Owner 6 was more likely to have type 2-5-2 and was also apparently clustered in space (Figure 1). Herds in Ownership 1 were also at increased risk of having type 2-5-2, and this was one of the large owners using the Boeringer-Ingelheim Ingelvac PRRS MLV™ vaccine. The pattern of space-time autocorrelation for RFLP type 2-5-2 indicated that diffuse clustering existed at > 20 km and 60 days separation of cases. Cases of this genotype did not exist closer than 5 km and 60 days from one another, and therefore the space-time K-function could not be estimated for the most proximal herds. In this study, vaccine strains were investigated to contrast findings with the patterns discovered for field strains. In that respect, it is important to note that space-time clustering existed for RFLP type 2-5-2, but the K-function plot suggested a pattern that could not be attributed to local spread among neighbouring herds. Because of the strong ownership effect and the indication of the effect of vaccine use, the detected autocorrelation was attributed to grouping of herd ownership in space.

The temporal rise and fall of RFLP type 1-4-2 (Figure 5) through the study period may reflect several possible scenarios. First, the outbreak of porcine circovirus type 2 associated disease (PCVAD) in Ontario in 2005 [36,37] that followed a reported short-lived outbreak of PRRSV in the winter of 2004–2005 [7] may have affected submission of samples to the laboratory. If herd veterinarians were making submissions to the AHL for PRRSV testing, but in fact the disease occurring was PCVAD, then more of the samples tested would be vaccine-type PRRSV, because wild-type PRRSV was not causing the disease. It is also plausible that these PCVAD outbreaks may have sparked an increase in the use of the Boeringer-Ingelheim Ingelvac PRRS ATP™ vaccine, which could have resulted in an increase in PRRSV RFLP-type 1-4-2 in the population. In addition, wild-type RFLP 1-4-2 virus was present in the Ontario swine population prior to the introduction of the PRRS ATP™ vaccine in the period between 1998 and 2000 [8].

Small owners were more likely than large owners to use a vaccine against PRRSV, but the large owners that did use a vaccine were more likely to choose the MLV™ vaccine than the ATP™ vaccine. This preferential use of PRRSV vaccine by one herd demographic could have affected the distribution of either of the two vaccine-type viruses in the population.

### Non-significant clusters

A reliable conclusion could be drawn only for statistically significant clusters. The exploration of the most-likely, non-significant clusters helped to reveal that spatial and space-time clusters could be qualitatively described in three ways: (i) clusters that were clearly due to herd links such as ownership or gilt source, (ii) clusters with no common links other than spatial proximity that was suggestive of local spread, (iii) clusters with no common links, but with spatial proximity that was too diffuse to be consistent with local spread. From a practical perspective, this result suggests that investigation of spatial and spatial-temporal clusters alone in epidemiological studies or outbreak investigations of PRRSV would not be sufficient to understand the origins of the clustering. Information regarding the animal-source and ownership connections between herds is necessary to explain the origin of some of the clusters. Significant clusters are also scarce in the present data. This might indicate that using a cluster-detection system for real-time surveillance may not be an adequate strategy to detect spread of PRRSV.

### Study design and limitations

A limitation of the study is inclusion of only 1 isolate of PRRSV per herd. It is understood that, in a herd co-infected with multiple PRRSV genotypes, some RFLP types are more likely to be detected by PCR than others [38]. This principle might have biased our results toward these genotypes. A second limitation is the use of laboratory submissions that were not incident cases. This was necessary because of the high prevalence of PRRSV in the population and the difficultly in showing that a reported incident case truly was the first occurrence of the disease in a herd. The importance of this limitation is reduced by use of a specific genotype of PRRSV as the case definition, because a specific genotype of PRRSV is more likely to be a new introduction than PRRSV in general. Finally, the use of diagnostic data for epidemiological investigations of this nature is problematic. The decision to submit a sample to a laboratory from a particular premises is also a critical decision, made by the herd veterinarian, that could affect this study. A decision to make a laboratory submission may have been related to the outcome (RFLP type) and exposure (location, ownership, animal source) and may therefore have lead to selection bias. The direction of this bias is difficult to conceptualize and might be differential. The motivation and the decisions surrounding diagnostic sample submission are currently not well understood and deserve further investigation.

## Conclusions

While the literature indicates that PRRSV can spread via aerosol between pig herds, strong evidence of this occurring in Ontario could not be detected in the present study despite using multiple analytical techniques to uncover patterns. The evidence pointed toward transmission of PRRSV occurring in this population by common sources of animals and other herd inputs.

This study showed that interpretation of spatial and space-time clusters, or space-time trends, specific PRRSV genotypes, was made possible by the availability of information about common animal sources and other connections between the study herds. Veterinary authorities and producer groups that aim to identify spatial and spatio-temporal clusters in PRRSV control programs should be aware that herd network information is important to gather. It should also be acknowledged that cluster detection might not be suitably sensitive for real-time surveillance of PRRSV outbreaks. In our study, clustering and space-time autocorrelation was detected for RFLP type 1-18-4. If gilt supplier information had been unavailable, the origin of these spatial patterns may have been misinterpreted as aerosol transmission. Conversely, the lack of common animal suppliers in the members of the spatial cluster detected for RFLP type 1-3-4 contributed to interpreting the cluster as a possible example of area spread. The aggregation of RFLP type 1-3-4, suggests that there are areas with dominant genotypes of PRRSV. Understanding that PRRSV genotypes may group geographically for long periods of time needs to be further investigated and could aid in the design of regional control programmes for PRRSV.

## Competing interests

The authors declare that they have no competing interests related to this publication.

## Author’s contributions

TR participated in the development, data collection, data analysis, interpretation and writing of the study. CD participated in the development, interpretation, and writing of the study. RF participated in development, interpretation, and writing of the study. SW participated in interpretation and writing of the study. BY participated in data collection and interpretation of the study. ZP participated in the development, data collection, data analysis, interpretation, and writing of the study. All authors have seen and approved the final manuscript.
